# p21^WAF1/CIP1^expression in stage I cutaneous malignant melanoma: its relationship with p53, cell proliferation and survival

**DOI:** 10.1038/sj.bjc.6690143

**Published:** 1999-02

**Authors:** J M Karjalainen, M Reinikainen, V-M Kosma

**Affiliations:** Department of Surgery, Kuopio University Hospital, PO Box 1777, Kuopio, FIN-70211, Finland; Department of Pathology and Forensic Medicine, University of Kuopio, PO Box 1627, Kuopio, FIN-70211, Finland; Department of Clinical Pathology, Kuopio University Hospital, PO Box 1777, Kuopio, FIN-70211, Finland

**Keywords:** malignant melanoma, prognosis, p21, p53, proliferation, immunohistochemistry

## Abstract

The expression of p21, p53 and proliferating cell nuclear antigen (PCNA) was analysed by immunohistochemistry in a consecutive series of 369 clinical stage I cutaneous malignant melanoma patients. Correlation of the detected expression levels with each other, with clinicopathological data and with melanoma survival were statistically evaluated. p21 expression was significantly associated with p53 and PCNA expression levels. In addition, high levels of p53 and PCNA were significantly interrelated. Tumour thickness, recurrent disease, high TNM category and older (≥ 55 years) age at diagnosis were inversely associated with p21 expression. Gender, bleeding, tumour thickness, Clark's level of invasion, TNM category and p53 index were all important predictors of both recurrence-free and overall survival of melanoma. In Cox's multivariate analysis including 164 patients with a complete set of data, only high tumour thickness and bleeding predicted poor recurrence-free survival (*P*= 0.0042 and 0.0087 respectively) or overall survival (*P*= 0.0147 and 0.0033 respectively). Even though elevated p21 expression may be associated with more favourable prognosis in clinical stage I cutaneous melanoma, our results suggest that cell cycle regulatory effects of p21 can be overcome by some other and stronger, partly yet unknown, mechanisms. 1999 Cancer Research Campaign


*British Journal of Cancer* (1999) **79**(5/6), 895–902
© 1999 Cancer Research Campaign
Article no. bjoc.1998.0143

### p21$^{WAF1/CIP1}$ expression in stage I cutaneous malignant melanoma: its relationship with p53, cell proliferation and survival

**JM Karjalainen[1,2], MJ Eskelinen[1], JK Kellokoski[2], M Reinikainen[2], EM Alhava[1] and V-M Kosma[2,3]**

[1]Department of Surgery, Kuopio University Hospital, PO Box 1777, FIN-70211 Kuopio, Finland; [2]Department of Pathology and Forensic Medicine, University of Kuopio, PO Box 1627, FIN-70211 Kuopio, Finland; [3]Department of Clinical Pathology, Kuopio University Hospital, PO Box 1777, FIN-70211 Kuopio, Finland

**Summary** The expression of p21, p53 and proliferating cell nuclear antigen (PCNA) was analysed by immunohistochemistry in a consecutive series of 369 clinical stage I cutaneous malignant melanoma patients. Correlation of the detected expression levels with each other, with clinicopathological data and with melanoma survival were statistically evaluated. p21 expression was significantly associated with p53 and PCNA expression levels. In addition, high levels of p53 and PCNA were significantly interrelated. Tumour thickness, recurrent disease, high TNM category and older (≥ 55 years) age at diagnosis were inversely associated with p21 expression. Gender, bleeding, tumour thickness, Clark's level of invasion, TNM category and p53 index were all important predictors of both recurrence-free and overall survival of melanoma. In Cox's multivariate analysis including 164 patients with a complete set of data, only high tumour thickness and bleeding predicted poor recurrence-free survival ($P$ = 0.0042 and 0.0087 respectively) or overall survival ($P$ = 0.0147 and 0.0033 respectively). Even though elevated p21 expression may be associated with more favourable prognosis in clinical stage I cutaneous melanoma, our results suggest that cell cycle regulatory effects of p21 can be overcome by some other and stronger, partly yet unknown, mechanisms.

**Keywords:** malignant melanoma; prognosis; p21; p53; proliferation; immunohistochemistry

Tumour suppressor *p21*$^{WAF1/CIP1}$ is the main downstream effector gene mediating p53-induced cell cycle arrest, up-regulated by normal wild-type *p53* (El-Deiry et al, 1993). In addition to direct transcriptional induction by the tumour suppressor *p53*, various other signals can induce *p21* expression in the absence of wild-type *p53* (Michieli et al, 1994; Zeng and El-Deiry, 1996). Harper et al (1993) showed that the ability of the *p21* gene product to arrest the cell cycle is based on its virtue to bind and inhibit active cyclin/CDK complexes needed in DNA replication and cell cycle progression. p21 can also inhibit DNA replication by directly binding to the proliferating cell nuclear antigen (PCNA) molecule, thus inhibiting the ability of DNA polymerase δ to extend new DNA chains but still allowing the DNA repair function to continue (Flores-Rozas et al, 1994; Waga et al, 1994; Podust et al, 1995).

In human melanoma cell lines, induction of *p21* is independent of wild-type p53 expression (Jiang et al, 1995; Vidal et al, 1995), and elevated expression of p21 is associated with melanoma differentiation, growth arrest and metastatic suppression (Jiang et al, 1995). However, the knowledge of p21 expression in clinical materials of cutaneous malignant melanoma is rather limited. Maelandsmo et al (1996) revealed a significant correlation between elevated p21 expression and increasing tumour thickness in superficially spreading melanoma, but no correlation between p21 and survival was found. Elsewhere, a significant overexpression of p21 has been demonstrated in primary and metastatic melanomas (Trotter et al, 1997).

As far as the authors are aware, no previous study has compared the relationships between p21, p53 and PCNA expression in primary stage I cutaneous melanoma. In the present study, we used immunohistochemistry to analyse the above-mentioned relationships in a cohort of 369 patients with long-term follow-up data. In addition, our aim was to analyse whether p21 protein levels are related to clinical data, histological parameters (Clark and Breslow levels) and prognosis.

### MATERIALS AND METHODS

### Patients

The retrospective study consists of a consecutive series of 369 clinical stage I cutaneous malignant melanoma patients with complete clinical and histopathological data available, who were diagnosed and treated in the district of Kuopio University Hospital, Finland, between 1974 and 1989. The clinical staging of all tumours was carried out according to UICC (UICC, 1987). Because of insufficient tumour material, pigment or technical artefacts, there were 267 valid immunostainings for p21, 284 for p53 and 219 for PCNA respectively. Patient records were reviewed and the pertinent clinical and histopathological data of the patients are shown in Table 1. The mean follow-up time of all 369 patients was 6.4 years (range 0.2–18 years). When the analysis was restricted to patients with p21, p53 or PCNA data, the mean follow-up times were 6.3 years (range 0.5–18 years), 6.3 years (range 0.5–18 years) or 7.2 years (range 0.5–18 years) respectively. The cause of death was obtained from the patient records and from the files of the Finnish Cancer Registry and General Statistical Office in Finland.

*Received 19 February 1998*
*Revised 21 May 1998*
*Accepted 4 June 1998*

*Correspondence to:* J Karjalainen, Department of Pathology and Forensic Medicine, University of Kuopio, PO Box 1627, FIN-70211 Kuopio, Finland

### Histology

The formalin-fixed, paraffin-embedded samples were sectioned at 5 μm and stained with haematoxylin and eosin (HE). The histological diagnosis was confirmed by reviewing 1–4 original sections of the primary tumour. Tumour thickness according to Breslow (1970) and level of invasion according to Clark et al (1969) were re-examined from the most representative slide by the same pathologist (VMK), unaware of the clinical data. In addition, the most representative block was selected to be cut into new 5-μm-thick sections for immunohistochemical studies.

### Immunohistochemistry

### *p21 and p53 stainings*

Sections were deparaffinized, rehydrated, washed twice for 5 min with distilled water and boiled in a microwave oven in citrate buffer (pH 6.0) for 5 × 5 min for antigen retrieval. Endogenous peroxidase activity was blocked by 5% hydrogen peroxide for 5 min, followed by washings for 2 × 3 min with distilled water and 2 × 5 min with phosphate-buffered saline (PBS) (pH 7.2). After eliminating non-specific staining with normal horse serum, the tissue sections were incubated overnight at 4°C with a p21-specific mouse monoclonal antibody (NCL-WAF-1, Novocastra Laboratories, UK) at a working dilution of 1:10. Samples were washed twice for 5 min with PBS and incubated for 30 min with biotinylated secondary antibody (Vectastain ABC Elite Kit, Vector Laboratories, CA, USA) in PBS. After two washings for 5 min in PBS, the sections were incubated for 40 min in preformed avidin–biotinylated–peroxidase complex solution. Samples were washed for 2 × 5 min with PBS, developed with diaminobenzidine tetrahydrochloride (DAB) substrate (Sigma, UK) for 5 min, slightly counterstained with Mayer's haematoxylin, dehydrated, cleared and mounted with DePex (BDH, Poole, UK).

The p53 protein was demonstrated by means of the same staining protocol. We used a monoclonal D07 (Dako, Denmark) antibody, known to be specific for both mutant and wild-type forms of the p53 protein (Vojtesek et al, 1992), at a working dilution of 1:1000. For antigen retrieval, the samples were boiled in a microwave oven twice for 5 min in citrate buffer (pH 6.0). In each batch, known p53- and p21-positive melanoma tumour samples were used as positive controls, and the same biopsy processed without the primary antibody was used as a negative control.

### *PCNA staining*

The staining protocol used in PCNA immunostaining has been described previously (Aaltomaa et al, 1993). In brief, 5-μm paraffin sections were deparaffinized and rehydrated. After blocking of endogenous peroxidase with 5% hydrogen peroxide and inhibition of non-specific staining with normal goat serum, the sections were incubated overnight with the PC10 (Dako) anti-PCNA monoclonal antibody diluted at 1:350 in PBS (pH 7.2) at 4°C. No antigen retrieval was used. Sections were washed twice for 5 min with PBS and incubated for 30 min with horse antimouse biotinylated secondary antibody (Vectastain ABC Elite Kit, Vector Laboratories, CA, USA) diluted at 1:200 in PBS (pH 7.2). After washing twice for 5 min in PBS, sections were incubated for 40 min in preformed avidin–biotin–peroxidase complex solution. The colour reaction was demonstrated with a chromogen (DAB) as described for p21. Normal human tonsil was used as a positive control, and the same biopsy processed without the primary antibody served as a negative control.

**Table 1** Clinical and histopathological data of 369 patients with stage I cutaneous malignant melanoma

| | |
|---|---|
| Gender | |
| Male | 178 |
| Female | 191 |
| Age (years) | |
| Mean (s.d.) | 55.5 (15.2) |
| Range | 19.0–89.7 |
| Anatomic site | |
| Head and neck | 64 |
| Trunk and perineum | 173 |
| Upper limbs | 58 |
| Lower limbs | 74 |
| Bleeding of the primary tumour | |
| Yes | 98 |
| No | 152 |
| Data not available | 119 |
| Growth of the primary tumour | |
| Yes | 200 |
| No | 38 |
| Data not available | 131 |
| Cause of death | |
| Malignant melanoma | 66 |
| Other | 46 |
| Alive | 257 |
| Recurrent disease | |
| Yes | 106 |
| No | 263 |
| Clark's level | |
| I | 21 |
| II | 68 |
| III | 106 |
| IV | 145 |
| V | 29 |
| Tumour thickness | |
| ≤0.75 mm | 82 |
| 0.76–1.50 mm | 88 |
| 1.51–4.0 mm | 120 |
| >4.0 mm | 50 |
| Not possible to analyse | 29 |
| TNM category | |
| pT1 or T2, N0, M0 | 174 |
| pT3, N0, M0 | 139 |
| pT4, N0, M0 | 56 |

### Scoring and quantitation of the immunoreactivities

All slides were evaluated with a dual-head microscope (field diameter 490 μm) by two observers (JMK and VMK for p21 and p53, MR and VMK for PCNA), who were unaware of the clinical outcome of the patients. For p21 and p53, the positivity was assessed as the percentage of positively stained tumour cell nuclei in the entire tumour area. The p21 positivity was scored as follows: 0 (< 1% positive), 1 (1–10% positive), 2 (10–20% positive) or 3 (> 20% positive). A semiquantitative grading was used for p53 staining, taking into account both staining intensity and proportion of positive tumour cells. Intensity was recorded from 0 (no cells positive) to 3 (strong staining). A staining index was then calculated by multiplying the fraction of stained nuclei (%) by the staining intensity (0–3). For statistical purposes, the tumours were divided according to this index into four groups: group 0 (0, $n$ = 91), group 1 (≤ 1, $n$ = 59), group 2 (1–10, $n$ = 83), and group 3 (> 10, $n$ = 51).

*© Cancer Research Campaign 1999*

**Table 2** Expression of p21, p53 and PCNA in stage I cutaneous malignant melanoma

| Immunostaining | *n* | % |
|---|---|---|
| p21 | | |
| 0 (<1%) | 44 | 17 |
| 1 (1–10%) | 103 | 39 |
| 2 (10–20%) | 58 | 22 |
| 3 (>20%) | 62 | 23 |
| Total | 267 | 100 |
| p53 index | | |
| 0 (0) | 91 | 32 |
| 1 (≤1) | 59 | 21 |
| 2 (1–10) | 83 | 29 |
| 3 (>10) | 51 | 18 |
| Total | 284 | 100 |
| PCNAtot | | |
| Low (≤35%) | 114 | 52 |
| High (>35%) | 105 | 48 |
| Total | 219 | 100 |

PCNAtot, PCNA expression in ten consecutive high-power fields; p53 index, (% of p53-positive tumour cells) × (staining intensity of p53).

**Table 3** Correlation between p21, p53 and PCNA expression and tumour thickness in stage I cutaneous malignant melanoma

| Variables | *n* | Spearman's correlation coefficient | Two-tailed significance |
|---|---|---|---|
| p21 vs. p53 index | 255 | 0.177 | 0.005 |
| p21 vs. PCNAtot | 181 | 0.368 | <0.00005 |
| p21 vs. tumour thickness | 251 | –0.164 | 0.009 |
| p53 index vs. PCNAtot | 194 | 0.296 | <0.00005 |
| p53 index vs. tumour thickness | 265 | 0.020 | 0.742 |
| PCNAtot vs. tumour thickness | 207 | –0.046 | 0.511 |

PCNAtot, PCNA expression in ten consecutive high power fields; p53 index, (% of p53-positive tumour cells) × (staining intensity of p53).

The PCNA positivity was scored as the fraction (%) of positively stained tumour cell nuclei evaluated in ten microscopic fields (diameter 490 µm, objective magnification 40×, corresponding to 0.194 $mm^2$ of neoplastic epithelium) from the area of the highest immunopositivity (PCNAtot) (Aaltomaa et al, 1993). The immunopositivity was further divided into two groups: low PCNAtot (≤ 35% of cells positive, $n$ = 114) and high PCNAtot (> 35% of cells positive, $n$ = 105).

A

B

C

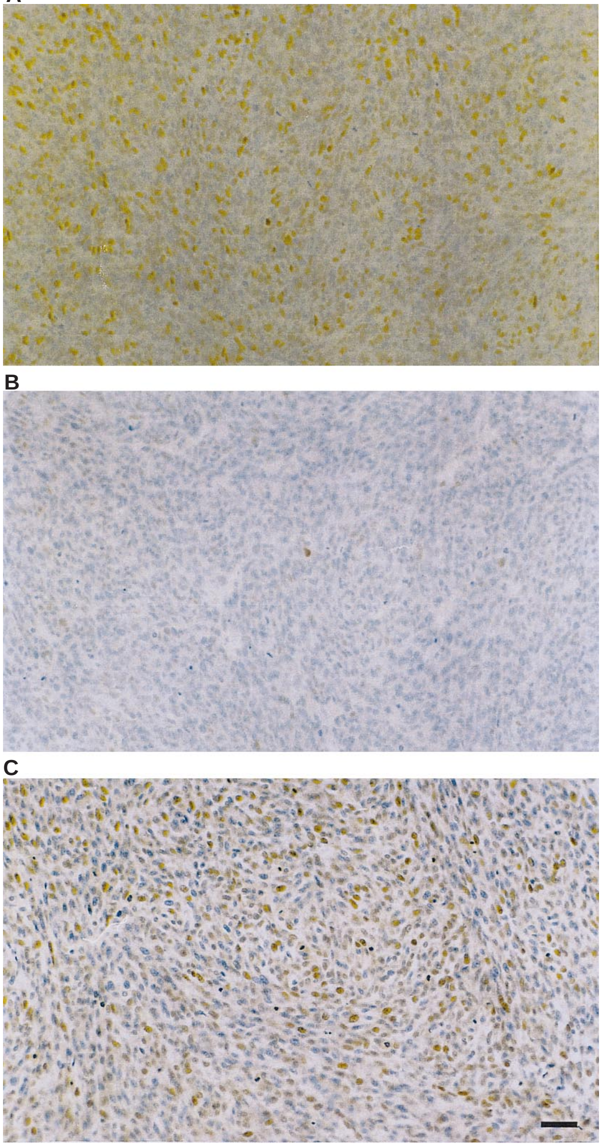

**Figure 1** Immunohistochemical stainings of cutaneous malignant melanoma. (**A**) Cutaneous malignant melanoma showing strong p21 immunopositivity. (**B**) An adjacent section showing only few p53-positive tumour cell nuclei. (**C**) PCNA immunostaining of the same specimen showing strong PCNA signal. Bar, 60 µm

### Statistical analyses

The SPSS-Win 7.5 program package was used in a PC computer for basic statistical calculations. First, the relationships (Spearman rank correlations) between p21, p53 and PCNA expression levels as well as tumour thickness were analysed using each parameter as a continuous variable. For all further statistical analyses, immunohistochemically determined parameters were categorized as previously described. The interrelationships between these immunohistochemical variables and their association with clinicopathological parameters were examined by contingency tables, which were further analysed by $\chi^2$ tests. Univariate survival analyses were based on the Kaplan–Meier method (log-rank analysis) (Kaplan and Meier, 1958). To assess the prognostic significance of dichotomic immunohistochemical variables (p21 expression, p53 index and PCNAtot) within each Breslow thickness subgroup, we made both univariate Kaplan–Meier and multivariate subset analyses within each Breslow category, as well as in tumours with Breslow thickness < 1.5 mm and > 1.5 mm. Multivariate survival analyses were carried out with the SPSS-Cox (Cox 1972) program package using the log likelihood ratio significance test in both forward and backward stepwise manner. Overall survival (OS) analysis included as an event only the deaths due to malignant melanoma. Deaths due to post-operative complications within 30 days were excluded. Recurrence-free survival (RFS) was defined as the time elapsed between the primary treatment and the recurrence of melanoma. For all statistical tests, a critical significance level of 5% was chosen. In Cox's multivariate

© Cancer Research Campaign 1999

**Table 4** Associations between p21 expression, p53 index and proliferation in stage I cutaneous malignant melanoma

| | p21 < 1% *n* (%) | p21 1–10% *n* (%) | p21 10–21% *n* (%) | p21 > 20% *n* (%) | $\chi^2$ | *P*-value |
|---|---|---|---|---|---|---|
| p53 index | | | | | | |
| 0 (0) | 23 (54) | 33 (35) | 12 (21) | 12 (20) | | |
| 1 (≤1) | 4 (9) | 21 (22) | 11 (19) | 17 (28) | | |
| 2 (1–10) | 3 (7) | 32 (34) | 22 (39) | 19 (32) | 5.826 | 0.016 |
| 3 (>10) | 13 (30) | 9 (9) | 12 (21) | 12 (20) | | |
| Total | 43 (100) | 95 (100) | 57 (100) | 60 (100) | | |
| PCNAtot | | | | | | |
| ≤35% | 21 (78) | 45 (65) | 17 (45) | 14 (30) | 21.68 | <0.00005 |
| >35% | 6 (22) | 24 (35) | 21 (55) | 33 (70) | | |
| Total | 27 (100) | 69 (100) | 38 (100) | 47 (100) | | |
| | **p53 index 0** *n* (%) | **p53 index ≤1** *n* (%) | **p53 index 1–10** *n* (%) | **p53 index > 10** *n* (%) | $\chi^2$ | *P*-value |
| PCNAtot | | | | | | |
| ≤35% | 47 (65) | 18 (46) | 25 (44) | 9 (35) | 9.225 | 0.002 |
| >35% | 25 (35) | 21 (54) | 32 (56) | 17 (65) | | |
| Total | 72 (100) | 39 (100) | 57 (100) | 26 (100) | | |

PCNAtot, PCNA expression in ten consecutive high power fields; p53 index, (% of p53-positive tumour cells) × (staining intensity of p53).

analysis, a removal limit of $P < 0.10$ was used as an additional inclusion criteria.

### RESULTS

### p21 expression

Of the 267 valid immunostainings for p21 analysis, 84% of the tumours showed at least 1% of their nuclei to express p21 protein. The distribution of p21 expression into different categories is shown in Table 2. The positive staining was remarkably confined to the tumour cell nuclei (Figure 1A). The detected p21 expression level was significantly related to p53 staining index and to proliferation assessed by PCNAtot (Tables 3 and 4), but p21 was also inversely related to p53 as shown in Table 4. In addition, reduced level of p21 expression was significantly associated with high tumour thickness ($\chi^2 = 5.76$, $P = 0.016$, $n = 267$; see also Table 3), recurrent disease ($\chi^2 = 4.26$, $P = 0.039$, $n = 267$), high TNM category ($\chi^2 = 4.92$, $P = 0.027$, $n = 267$), and older (≥ 55 years) age at the time of diagnosis ($\chi^2 = 4.16$, $P = 0.041$, $n = 267$) (other data not shown). No significant associations were observed between the p21 expression level and gender or bleeding (data not shown).

### p53 expression

There were 284 immunostainings available for p53 analysis, of which 68% showed detectable levels (> 0%) of protein accumulation. Only about 20% of the tumours showed p53 immunopositivity in more than 5% of the tumour cell nuclei, and the p53 staining index was over 10 in 18% of the cases (Table 2). The p53 staining was confined mostly to the tumour cell nuclei (Figure 1B), but in 14% of the cases cytoplasmic staining was also detectable.

There was a strong statistically significant association between high p53 staining index and high proliferation (PCNAtot) (Tables 3 and 4). Of the p53 staining index-negative tumours, 65% showed low PCNAtot (≤ 35%) expression levels, whereas only 35% of the tumours with high p53 staining index (> 10) showed low PCNAtot levels ($\chi^2 = 9.225$, $P = 0.002$; Table 4). The p53 staining index and tumour thickness were not statistically interrelated (Table 3), whereas a significant association was observed between the high p53 staining index and recurrent disease ($\chi^2 = 5.853$, $P = 0.016$; other data not shown).

### PCNA expression

Detectable levels (> 0%) of PCNA positivity were observed in 99.5% of the cases, and the positive tumour cell nuclei were usually evenly distributed over the entire tumour area (Figure 1C). PCNAtot was related neither to tumour thickness nor any other clinicopathological variable (data not shown).

### Univariate survival analysis

During the follow-up (mean 6.4 years), 106 patients (29%) had a recurrence, 66 patients (18%) died of melanoma and 46 patients (13%) died of other causes. The crude 5-year survival rate of the patients was 78%. The 5-year overall survival (OS) rate of the patients was 85%, and the 5-year recurrence-free survival (RFS) rate was 76%. The corresponding survival rates among the subgroups with valid p21, p53 and PCNA immunostainings were 83%, 85% and 84% for OS and 73%, 75% and 74% for RFS respectively. Significant clinicopathological factors predicting OS as well as RFS in univariate analysis were gender, bleeding, tumour thickness, Clark's level of invasion and TNM category (Table 5).

We found no statistically significant relationship between the expression level of p21 and OS or RFS of patients with melanoma, even though there was a trend that the patients with highly p21 positive (> 20%) tumours had a better 5-year RFS (79%) than the patients with p21-negative tumours (73%) ($P = 0.076$) (Table 5). Further on, p21 expression was not associated with OS or RFS within any tumour thickness subgroup. The high (> 10) p53 staining index was a significant predictor of poor OS ($P = 0.0086$) and poor RFS ($P = 0.0112$) in all patients (Table 5), as well as in patients with tumour thickness > 1.5 mm ($P = 0.001$ for OS and $P = 0.019$ for RFS) (other data not shown). We also tested the prognostic significance of cytoplasmic p53 staining, but it had no impact on survival

 © Cancer Research Campaign 1999

**Table 5** Clinical, histological and immunohistochemical factors related to survival in stage I cutaneous malignant melanoma

| Category (variable) | No. of patients | Recurrence-free 5 years (RFS) (%) | *P*-value* | Surviving at 5 years (%) | *P*-value* |
|---|---|---|---|---|---|
| Gender | | | 0.0113 | | 0.0056 |
| Male | 178 | 75 | | 79 | |
| Female | 191 | 89 | | 89 | |
| Bleeding | | | <0.00005 | | 0.0002 |
| Yes | 98 | 74 | | 77 | |
| No | 152 | 90 | | 92 | |
| Data not available | 119 | 79 | | 83 | |
| Clark's level | | | <0.00005 | | <0.00005 |
| I | 21 | 94 | | 94 | |
| II | 68 | 98 | | 98 | |
| III | 106 | 90 | | 92 | |
| IV | 145 | 71 | | 76 | |
| V | 29 | 65 | | 65 | |
| Tumour thickness | | | <0.00005 | | <0.00005 |
| ≤0.75 mm | 82 | 97 | | 97 | |
| 0.76–1.50 mm | 88 | 90 | | 93 | |
| 1.51–4.0 mm | 120 | 74 | | 78 | |
| >4.0 mm | 50 | 57 | | 63 | |
| TNM category | | | | | |
| pT1 or T2, N0, M0 | 174 | 95 | <0.00005 | 96 | <0.00005 |
| pT3, N0, M0 | 139 | 74 | | 78 | |
| pT4, N0, M0 | 56 | 60 | | 65 | |
| p21 expression | | | 0.28 | | 0.78 |
| <1% | 44 | 73 | | 91 | |
| 1–10% | 103 | 73 | | 84 | |
| 10–20% | 58 | 74 | | 85 | |
| >20% | 62 | 79 | 0.0762 | 84 | |
| p53 index | | | 0.0112 | | 0.0086 |
| 0 | 91 | 86 | | 94 | |
| ≤1 | 59 | 79 | | 88 | |
| 1–10 | 83 | 71 | | 82 | |
| >10 | 51 | 55 | 0.0015 | 68 | 0.0013 |
| PCNAtot | | | 0.098 | | 0.55 |
| ≤35% | 114 | 81 | | 87 | |
| >35% | 105 | 66 | | 80 | |

PCNAtot, PCNA expression in ten consecutive high power fields; p53 index, (% of p53-positive tumour cells) × (staining intensity of p53); *log-rank analysis.

(data not shown). The patients with actively proliferating tumours assessed by high (> 35%) PCNAtot expression had a worse 5-year RFS compared with the patients with reduced (≤ 35%) levels, but the difference between the survival curves was not signicant ($P$ = 0.098) (Table 5). However, high PCNAtot expression predicted poor RFS in patients with tumour thickness > 1.5 mm ($P$ = 0.027) (other data not shown). In OS, there was no statistical difference between the survival curves in tumours of different PCNAtot categories ($P$ = 0.55) (Table 5). To determine whether the actively proliferating tumours might have different survival according to their p21 positivity, we separated the tumours by their p21 expression level and proliferation status. Indeed, the combination of reduced p21 and increased PCNA expression was a marker for poor RFS. However, the difference was only of limited significance ($P$ = 0.0636) (other data not shown).

### Multivariate survival analysis

There were 164 patients with a complete set of data available for a multivariate Cox's analysis. This included the significant clinicopathological variables of the univariate analysis (gender, bleeding, tumour thickness, Clark's level of invasion) completed by the immunohistochemically determined variables (p21 expression, p53 staining index and PCNAtot). The pT category (consisting of Clark's level of invasion and tumour thickness) was excluded from the final model. Only tumour thickness ($P$ = 0.0042) and bleeding ($P$ = 0.0087) predicted poor RFS as well as poor OS ($P$ = 0.0147 for tumour thickness and $P$ = 0.0033 for bleeding) in Cox's analysis (other data not shown). In the subset Cox's analyses according to tumour thickness, high p53 index (overall $P$ = 0.038) and bleeding (overall $P$ = 0.042) were independently associated with poor OS in patients with > 1.5-mm-thick tumours ($n$ = 96). No other combination proved to be of independent prognostic value in OS or RFS analyses (other data not shown).

### DISCUSSION

In the previous studies with clinical material, p21 expression has correlated with tumour proliferation and differentiation either in a *p53*-dependent (Doglioni et al, 1996; Harada et al, 1997; Wakasugi et al, 1997) or in a *p53*-independent pathway (DiGiuseppe et al, 1995; Tron et al, 1996; Yasui et al, 1996; Nadal et al, 1997). So far,

*© Cancer Research Campaign 1999* 

only one clinical study has compared p21 expression with p53 in cutaneous malignant melanoma (Maelandsmo et al, 1996). Consequently, the prognostic role of p21 expression in cutaneous malignant melanoma has not yet been defined.

The majority (84%) of the tumours in our material showed at least 1% of their nuclei to express p21 protein. This result is comparable with the study by Maelandsmo et al (1996), in which 69% of the primary melanomas and 57% of the metastases were p21 positive. We found that thick tumours and tumours with high pT category express significantly lower levels of p21 protein. This observation contradicts the result of Maelandsmo et al (1996) in superficially spreading melanomas, but supports the hypothesis that loss of p21 function may be associated with melanoma progression and with metastatic phenotype (Jiang et al, 1995; Maelandsmo et al, 1996). The trend of reduced p21 expression in recurrent disease further suggests that *p21* may have a role in melanoma growth suppression. Additional support for this comes from the univariate survival analysis, in which tumours with high (> 20%) levels of p21 expression had a little better 5-year RFS (79%) than the p21-negative tumours (73%). However, this difference was not significant either in univariate or in multivariate survival analysis, and this compares well with the results of Maelandsmo et al (1996).

In line with the observations in breast cancer (Diab et al, 1997), we found a significant association between the p21 and p53 expression levels (Tables 3 and 4). This suggests a *p53*-independent induction of *p21* consistent with the findings in cutaneous melanoma cell lines (Jiang et al, 1995; Vidal et al, 1995) or in cutaneous melanoma in vivo (Maelandsmo et al, 1996). Unfortunately, the method used in the present study didn't allow us to exclude other possibilities for our observation, such as stabilization of wt-p53 (Wynford-Thomas, 1992; Hall and Lane, 1994), exclusion of the protein into cytoplasm (Moll et al, 1992) or *p21* activation by *p53* despite underlying *p53* mutation (Ory et al, 1994; Harada et al, 1997).

Comparative studies have shown that immunohistochemical detection of p53 is a good approximation of its real mutation (and subsequent dysfunction) rate (Melhem et al, 1995; Nishio et al, 1996). Based on this, the observation of inverse correlation between p21 and p53 immunopositivities would suggest a *p53*-dependent mechanism of *p21* activation. Indeed, we found several patients with an inverse correlation between p21 and p53 expression (Table 4). In addition, there was a subgroup of patients with concomitant lack of p53 and p21 expression (Table 4). It is possible that in some of those tumours D07 antibody did not recognize all mutated forms of p53 protein (Borresen et al, 1991). Moreover, other mechanisms than mutation may cause *p53* dysfunction, including complexing with viral oncoproteins or with mdm-2 protein (Gelsleichter et al, 1995). Thus, the relationship between p21 expression and *p53* is complex and seems to be tumour-type specific.

Highly variable results of p53 immunoreactivity in primary cutaneous melanoma (Weiss et al, 1995; Talve et al, 1996) have been explained by differences in staining protocols, antibodies, tumour materials (Talve et al, 1996) or cut-off levels used for scoring (Gelsleichter et al, 1995). In our study, p53 immunopositivity was observed in 68% of the samples, which is a clearly higher p53 expression level compared with the studies in which the same antibody without antigen retrieval was used (Lassam et al, 1993; Ro et al, 1993). In contrast, the fraction of immunopositivity in our material was very low, only about 20% of the tumours showing p53 signal in more than 5% of the tumour cell nuclei. This compares well with reports in which D07 antibody has been used in human cutaneous malignant melanoma (Sparrow et al, 1995; Talve et al, 1996). A high p53 staining index was significantly associated with poor RFS and OS in the univariate analyses, but lost its significance in the multivariate analysis of all patients. However, within the subgroup of patients with thick (>1.5 mm) tumours, already known to have an impaired survival, p53 index independently separated those patients into different prognostic groups. Most studies to date have shown either no correlation (Sparrow et al, 1995; Weiss et al, 1995; Talve et al, 1996) or, like our study, an inverse (Yamamoto et al, 1995; Vogt et al, 1997) correlation between the p53 expression and outcome of human cutaneous malignant melanoma.

The high proliferation (PCNAtot) rate was related to the increased p21 expression, which parallels the results in cutaneous melanoma (Trotter et al, 1997) and in cutaneous squamous cell carcinoma (Tron et al, 1996), but is in contrast to findings in other neoplasms (Nadal et al, 1997) or in normal tissues (El-Deiry et al, 1995; Doglioni et al, 1996). The possible explanations of our result may be either an abnormal function of p21 (Trotter et al, 1997) or that part of the tumour cells may have become refractory to inhibitory signals from *p21* (Yasui et al, 1996). It is also possible that the tumour cells may up-regulate *PCNA* expression as a response to elevated p21 expression levels to stay in the proliferative compartment (Waga et al, 1994). In contrast, the elevated p21 expression level may also be the result of a feedback mechanism designed to halt proliferation (Jung et al, 1995).

The cell cycle regulatory effect of p21 interaction with PCNA seems to be mediated only by large excess of p21 (Flores-Rozas et al, 1994; Podust et al, 1995). With respect to this, we further subdivided both PCNAtot categories into four groups according to their p21 expression level. In the actively proliferating tumours (PCNAtot >35%), high p21 positivity approached limited statistical significance as a marker of favourable univariate RFS ($P$ = 0.0636). Our finding may, thus, support the observation of tumour growth inhibitory function of *p21* in a p21–PCNA interaction manner (Flores-Rozas et al, 1994; Waga et al, 1994; Podust et al, 1995).

There are only few reports on p53 expression with respect to PCNA expression or other proliferation markers (Gelsleichter et al, 1995; Naresh et al, 1997). Gelsleichter et al (1995) found a positive correlation between p53 overexpression and MIB-1 expression in primary melanoma, whereas no association was found between p53 and PCNA expression levels. We found a positive correlation between high PCNAtot positivity and increased p53 index as did Naresh et al (1997) in Hodgkin's disease. This interesting result is in accordance with a suggestion that wt-*p53* down-regulates *PCNA* expression, whereas the mutant form up-regulates *PCNA* by activating its promoter (Deb et al, 1992). The progression of a malignant tumour may also result in increasing numbers of cells with abnormalities in DNA synthesis and subsequently in high levels of p53 and p21. To overcome their inhibitory effects on the cell cycle, the PCNA levels may be kept higher by independent mechanisms (Naresh et al, 1997).

In conclusion, our results suggest that there are both p53-independent and *p53*-dependent pathways of *p21* induction in cutaneous malignant melanoma. Immunohistochemically detected positive p53 (mutation or protein stabilization) was the only prognostically significant protein associated with poor disease outcome, especially within thick tumours. Even though elevated p21 expression may indicate favourable prognosis in clinical stage I cutaneous melanoma, it is probable that the growth inhibitory

© Cancer Research Campaign 1999

effects of *p21* can be overcome by some other and stronger, partly yet unknown, mechanisms. Such mechanisms could include interaction with inhibitory proteins (Gadd45) (Chen et al, 1995), cyclin D, E2F (Hiyama et al, 1997), or with other molecules (AP-2) (Zeng et al, 1997).

### ACKNOWLEDGEMENTS

This study has been supported by grants from the Cancer Fund of North Savo (Savon Syöpärahasto), the Culture Fund of Finland and by EVO-funding of Kuopio University Hospital. The authors thank Ms Merja Fali, Ms Seija Haatanen and Ms Aija Parkkinen for their skilful technical assistance. The invaluable statistical assistance of Ms Pirjo Halonen is also acknowledged.

### REFERENCES

Aaltomaa S, Lipponen P, Papinaho S and Syrjänen K (1993) Proliferating-cell nuclear antigen (PC10) immunolabelling and other proliferation indices as prognostic factors in breast cancer. *J Cancer Res Clin Oncol* **119**: 288–294

Borresen AL, Hovig E, Smith Sorensen B, Malkin D, Lystad S, Andersen TI, Nesland JM, Isselbacher KJ and Friend SH (1991) Constant denaturant gel electrophoresis as a rapid screening technique for p53 mutations. *Proc Natl Acad Sci USA* **88**: 8405–8409

Breslow A (1970) Thickness, cross-sectional areas and depth of invasion in the prognosis of cutaneous melanoma. *Ann Surg* **12**: 902–908

Chen I-T, Smith ML, O'Connor PM and Fornace AJ (1995) Direct interaction of Gadd45 with PCNA and evidence for competitive interaction of Gadd45 with $p21^{Wafl/Cip1}$ with PCNA. *Oncogene* **11**: 1931–1937

Clark Jr WH, From L, Bernardino EA and Mihm MC (1969) The histogenesis and biologic behaviour of primary human malignant melanomas of the skin. *Cancer Res* **29**: 705–726

Cox DR (1972) Regression models and life tables with discussion. *J Stat Soc B* **34**: 187–192

Deb S, Jackson CT, Subler MA and Martin DW (1992) Modulation of cellular and viral promoters by mutant human p53 proteins found in tumour cells. *J Virol* **66**: 6164–6170

Diab SG, Yu YY, Hilsenbeck SG, Allred DC and Elledge RM (1997) WAF1/CIP1 protein expression in human breast tumors. *Breast Cancer Res Treat* **43**: 99–103

DiGiuseppe JA, Redston MS, Yeo CJ, Kern SE and Hruban RH (1995) p53-independent expression of the cyclin-dependent kinase inhibitor p21 in pancreatic carcinoma. *Am J Pathol* **147**: 884–888

Doglioni C, Pelosio P, Laurino L, Macri E, Meggiolaro E, Favretti F and Barbareschi M (1996) p21/WAF1/CIP1 expression in normal mucosa and in adenomas and adenocarcinomas of the colon: its relationship with differentiation. *J Pathol* **179**: 248–253

El-Deiry WS, Tokino T, Velculescu VE, Levy DB, Parsons R, Trent JM, Lin D, Mercer WE, Kinzler KW and Vogelstein B (1993) WAF1, a potential mediator of p53 tumour suppression. *Cell* **75**: 817–825

El-Deiry WS, Tokino T, Waldman T, Oliner JD, Velculescu VE, Burrell M, Hill DE, Healy E, Rees JL, Hamilton SR, Kinzler KW and Vogelstein B (1995) Topological control of $p21^{WAF1/cip1}$ expression in normal and neoplastic tissues. *Cancer Res* **55**: 2910–2919

Flores-Rozas H, Kelman Z, Dean FB, Pan ZQ, Harper JW, Elledge SJ, O'Donnell MO and Hurwitz J (1994) Cdk-interacting protein I directly binds with proliferating cell nuclear antigen and inhibits DNA replication catalyzed by the DNA polymerase delta holoenzyme. *Proc Natl Acad Sci USA* **91**: 8655–8659

Gelsleichter L, Gown AM, Zarbo RJ, Wang E and Coltera MD (1995) P53 and mdm-2 expression in malignant melanoma: an immunohistochemical study of expression of p53, mdm-2, and markers of cell proliferation in primary versus metastatic tumours. *Modern Pathol* **8**: 530–535

Hall PA and Lane DP (1994) p53 in tumour pathology: can we trust immunohistochemistry? – Revisited! *J Pathol* **172**: 1–4

Harada N, Gansauge S, Gansauge F, Gause H, Shimoyama S, Imaizumi T, Mattfeld T, Schoenberg MH and Beger HG (1997) Nuclear accumulation of p53 correlates significantly with clinical features and inversely with the expression of the cyclin-dependent kinase inhibitor $p21^{WAF1/CIP1}$ in pancreatic cancer. *Br J Cancer* **76**: 299–305

Harper JW, Adami GR, Wei N, Keyomarsi K and Elledge SJ (1993) The p21-Cdk-interacting protein Cip1 is a potent inhibitor of $G_1$ cyclin-dependent kinases. *Cell* **75**: 805–816

Hiyama H, Iavarone A, LaBaer J and Reeves SA (1997) Regulated ectopic expression of cyclin D1 induces transcriptional activation of the cdk inhibitor p21 gene without altering cell cycle progression. *Oncogene* **14**: 2533–2542

Jiang H, Lin J, Su ZZ, Herlyn M, Kerbel RS, Weissman BE, Welch DR and Fisher PB (1995) The melanoma differentiation-associated gene mda-6, which encodes the cyclin-dependent kinase inhibitor p21, is differentially expressed during growth, differentiation and progression in human melanoma cells. *Oncogene* **10**: 1855–1864

Jung JM, Bruner JM, Ruan S, Langford LA, Kyritsis AP, Kobayashi T, Levin VA and Zhang W (1995) Increased levels of p21WAF1/Cip1 in human brain tumors. *Oncogene* **11**: 2021–2028

Kaplan EL and Meier P (1958) Nonparametric estimation from incomplete observations. *J Am Stat Assoc* **53**: 457–481

Lassam NJ, From L and Kahn HJ (1993) Overexpression of p53 is a late event in the development of malignant melanoma. *Cancer Res* **53**: 2235–2238

Maelandsmo GM, Holm R, Fodstad Ø, Kerbel RS and Florenes VA (1996) Cyclin kinase inhibitor $p21^{WAF1/CIP1}$ in malignant melanoma. *Am J Pathol* **149**: 1813–1822

Melhem MF, Law JC, El-Ashmawy L, Johnson JT, Landreneau RJ, Srivastava S and Whiteside TL (1995) Assessment of sensivity and specifity of immunohistochemical staining of p53 in lung and head and neck cancers. *Am J Pathol* **146**: 1170–1177

Michieli P, Chedid M, Lin D, Pierce JH, Mercer WE and Givol D (1994) Induction of WAF1/CIP1 by a p53-independent pathway. *Cancer Res* **54**: 3391–3395

Moll UM, Riou G and Levine AJ (1992) Two distinct mechanisms alter p53 in breast cancer: mutation and nuclear exclusion. *Proc Natl Acad Sci USA* **89**: 7262–7266

Nadal A, Jares P, Cazorla M, Fernandez PL, Sanjuan X, Hernandez L, Pinyol M, Aldea M, Mallofre C, Muntane J, Traserra J, Campo E and Cardesa A (1997) $p21^{WAF1/Cip1}$ expression is associated with cell differentiation but not with p53 mutations in squamous cell carcinomas of the larynx. *J Pathol* **183**: 156–163

Naresh KN, O'Conor GT, Soman CS, Johnson J, Advani SH, Magrath IT and Bhatia KG (1997) A study of p53 protein, proliferating cell nuclear antigen, and p21 in Hodgkin's disease at presentation and relapse. *Hum Pathol* **28**: 549–555

Nishio M, Koshikawa T, Kuroishi T, Suyama M, Uchida K, Takagi Y, Washimi O, Sugiura T, Ariyoshi Y, Takahashi T, Ueda R and Takahashi T (1996) Prognostic significance of abnormal p53 accumulation in primary, resected non-small-cell lung cancers. *J Clin Oncol* **14**: 497–502

Ory K, Legros Y, Auguin C and Soussi T (1994) Analysis of the most representative tumour-derived p53 mutants reveals that changes in protein conformation are not correlated with loss of transactivation or inhibition of cell proliferation. *EMBO J* **13**: 3496–3504

Podust VN, Podust LM, Goubin F, Ducommun B and Hubscher U (1995) Mechanism of inhibition of proliferating cell nuclear antigen-dependent DNA synthesis by the cyclin-dependent kinase inhibitor p21. *Biochemistry* **34**: 8869–8875

Ro YS, Cooper PN, Lee JA, Quinn AG, Harrison D, Lane D, Horne CH, Rees JL and Angus B (1993) p53 protein expression in benign and malignant skin tumours. *Br J Dermatol* **128**: 237–241

Sparrow LE, English DR, Heenan PJ, Dawkins HJ and Taran J (1995) Prognostic significance of p53 over-expression in thin melanomas. *Melanoma Res* **5**: 387–392

Talve L, Kainu J, Collan Y and Ekfors T (1996) Immunohistochemical expression of p53 protein, mitotic index and nuclear morphometry in primary malignant melanoma of the skin. *Pathol Res Pract* **192**: 825–833

Tron VA, Tang L, Yong WP and Trotter MJ (1996) Differentiation-associated overexpression of the cyclin-dependent kinase inhibitor $p21^{waf-1}$ in human cutaneous squamous cell carcinoma. *Am J Pathol* **149**: 1139–1146

Trotter MJ, Tang L and Tron VA (1997) Overexpression of the cyclin-dependent kinase inhibitor $p21^{waf-1/CIP1}$ in human cutaneous malignant melanoma. *J Cutan Pathol* **24**: 265–271

UICC (1987) *TNM Classification of Malignant Tumours*, 4th edn., Springer-Verlag, Berlin; pp. 88–90.

Vidal MJ, Loganzo Jr F, de Oliveira AR, Hayward NK and Albino AP (1995) Mutations and defective expression of the WAF1 p21 tumour-suppressor gene in malignant melanomas. *Melanoma Res* **5**: 243–250

Vogt T, Zipperer KH, Vogt A, Holzel D, Landthaler M and Stolz W (1997) p53-protein and Ki-67-antigen expression are both reliable biomarkers of prognosis in thick stage I nodular melanomas of the skin. *Histopathology* **30**: 57–63

© Cancer Research Campaign 1999

Vojtesek B, Bontek J, Midgley CA and Lane DP (1992) An immunochemical analysis of human p53: new monoclonal antibodies and epitope mapping using recombinant p53. *J Immunol Methods* **51**: 237–244
Waga S, Hannon GJ, Beach D and Stillman B (1994) The p21 inhibitor of cyclin-dependent kinases controls DNA replication by interaction with PCNA. *Nature* **369**: 574–578
Wakasugi E, Kobayashi T, Tamaki Y, Ito Y, Miyashiro I, Komoike Y, Takeda T, Shin E, Takatsuka Y, Kikkawa N, Monden T and Monden M (1997) p21(Waf1/Cip1) and p53 protein expression in breast cancer. *Am J Clin Pathol* **107**: 684–691
Weiss J, Heine M, Körner B, Pilch H and Jung EG (1995) Expression of p53 protein in malignant melanoma: clinicopathological and prognostic implications. *Br J Dermatol* **133**: 23–31
Wynford-Thomas D (1992) P53 in tumour pathology: can we trust immunohistochemistry? *J Pathol* **166**: 329–330
Yamamoto M, Takahashi H, Saitoh K, Horikoshi T and Takahashi M (1995) Expression of the p53 protein in malignant melanomas as a prognostic indicator. *Arch Dermatol Res* **287**: 146–151
Yasui W, Akama Y, Kuniyasu H, Yokozaki H, Semba S, Shimamoto F and Tahara E (1996) Expression of cyclin-dependent kinase inhibitor p21WAF1/CIP1 in non-neoplastic mucosa and neoplasia of the stomach: relationship with p53 status and proliferative activity. *J Pathol* **180**: 122–128
Zeng Y-X and El-Deiry WS (1996) Regulation of $p21^{WAF1/CIP1}$ expression by p53-independent pathways. *Oncogene* **12**: 1557–1564
Zeng Y-X, Somasundaram K and El-Deiry WS (1997) AP2 inhibits cancer cell growth and activates $p21^{WAF1/CIP1}$ expression. *Nature Genet* **15**: 78–82

*© Cancer Research Campaign 1999*
